# Consequences of Decay of the Teeth

**Published:** 1870-11

**Authors:** 


### Editorial.

### CONSEQUENCES OF DECAY OF THE TEETH.

In the following pages we shall attempt to present, though briefly, some of the results or consequences of decay of the teeth; and if by this means any shall be stimulated to a higher appreciation of their value and importance in the animal economy, this writing will not be wholly in vain.

The first result of caries of the teeth is partial or total loss of the organ; and so far as this is accomplished it is permanent. Nature makes no restoration or repair; many tissues and organs of the body, when lacerated, broken, or even when partially destroyed, will be repaired, or even reproduced. With the teeth no such effort is ever exercised, at least in the direction of restoration. The loss of the teeth by many is regarded as a matter of small moment. Such a feeling, however, arises from a total want of appreciation of the teeth and the very important functions they perform. Others place some value upon them as objects of beauty, caring nothing for them in any other respect; with such persons only those teeth that are exposed to view are valued; those teeth that are really of the greater importance are accounted of little value, and so receive little or no attention. Few if any place a proper estimate upon the teeth. This is clearly shown by the very general want of proper care of them. The loss of the teeth, with all its train of evils, is permanent. No substitution can completely fill any offices of the natural teeth.

The loss occasioned by the destructive process, when small in extent, may be repaired by art, using for the substitution a wholly foreign substance, so that in some cases there will be no interruption in any of their work. But in numberless other cases, when all has been accomplished that the highest art and skill can do, not only is there more or less interruption in the proper exercise of the functions, but there is a constant, persistent fear of evil to come, which arises from the very frequent realization of the thing anticipated, and notwithstanding all the pledges and promises that may be made in reference to permanency, the intelligent victim knows that a breach has been made in the wall, and that all attempts at reparation are but patch work, and is wanting in some, at least, of the elements of stability. Teeth that have been decayed though but little and repaired are always objects of solicitude to the appreciative possessor, even when no present inconvenience is experienced. But how often is it that after even the most skillful operations, annoyance is experienced, either constant or periodical, which will always occasion interruption in mastication.

But where there is the entire loss of several teeth from the arch, the infraction in every respect is of a more serious character, making an inroad upon every function of the teeth. The character of this infraction will be modified by the number of teeth destroyed and the location in the arch from which they are taken; the loss of the molars making the greatest breach in mastication; that of the incisors proving the greater obstacle to distinct and accurate enunciation of speech.

In all cases, with very rare exceptions, the result of dental caries is the entire destruction of the teeth affected, without the intervention of some aid in addition to that of nature. Were there a large number or any considerable proportion of the cases arrested short of the entire destruction of the tooth, at least its crown, the fearful importance that attaches to this subject would be somewhat lessened. Its frequency and fatality make it a subject of intense interest.

Another very common result of decay of the teeth is pain. In perhaps two-thirds of the cases there is more or less pain experienced, and this is frequently very soon. There are three points from which, and three distinct periods in the progress of the affection when pain is manifested.

The first is presented by an exalted sensitiveness of the dentine. This is usually exhibited in the most intense form in the incipient stages of decay. From this condition, however, pain is only experienced by mechanical contact, chemical action or thermal changes. Sensitive dentine, however highly it may be affected, is not capable of giving pain, except by the influence of outside impressions, and when these are removed it ceases and does not again recur till the irritant is present.

This affection of the dentine is most intense in the incipient stages of decay. There is very great variety in its manifestation, both in respect to degree and location. It is some times confined to the periphery of the dentine; at other times or in other cases it pervades the whole of the dentine, exercising an irritating influence upon the pulp, even to the extent of high inflammatory action; to such an extent does this some times proceed that death of the pulp occurs long before its exposure by decay.

As a result of decay of the teeth, pain of a very different character is experienced upon exposure of the pulp to the action of external irritants. The pain thus occasioned is usually intense in degree, persistant and constant in continuance, and often so severe as to render the victim incapable of rational self control.

The simple exposure of this tissue, which nature designed should be ever completely inclosed within the walls of its bony chamber, is usually quite sufficient to occasion disease and pain. This pain may be confined to the tooth pulp itself, or it may extend indefinitely out from that point and involve the surrounding parts.

The keen, sharp pain resulting from a highly inflamed tooth pulp has scarcely an equal by that presented in any other organ or tissue of the body. All this will result from active inflammation of the pulp, and from this acute state it may pass to one of a chronic character, and so for a long time be persistently annoying to the patient. There may be from it in this condition a discharge of pus, or it may assume a hypertrophied condition, enlarging so as to protrude through the orifice of exposure into the decayed cavity, and to such an extent does this some times occur that a large cavity of decay is filled with the fungus, and even protruding from it. And when this tissue becomes devitalized, the end is not yet, so far as pain and suffering is concerned; for very frequently following closely upon the heels of this devitalization, disease of the periosteum—the investing membrane of the root of the tooth—occurs, with a whole train of afflictions, pains and agonies, that refused to be conquered until the entire removal of the offending organ is effected. Inflammation, suppuration, and the total disorganization of the periosteum, are frequent results in the train that follows decay of the teeth. In this ruin is too often involved the affection, disease and death of the contiguous parts, both the soft and hard. Very often indeed do the gums and mucous membrane become involved, and that in a great variety of phases; from that of simple irritation and inflammation to a breaking down and a wasting away of the tissue, to the extent of its entire removal from the roots of the teeth; and from the occurrenee of the most simple benign tumor to that of the most malignant carcinomatous character, which when definitely developed usually proves speedily fatal. Not less striking and fearful are the effects upon the bony parts, causing solution and wasting away, or death and exfoliation, or the production of bony tumors of both malignant and non-malignant character, the results of which will be quite as serious as though in the soft parts, and perhaps more difficult to eradicate.

The transfer of affections from the roots and sockets of the teeth to the antrum is a matter fraught with very grave interest, both to the one who has it and to him who treats it. Affections of this cavity are often, even when arising from very simple causes, exceedingly difficult to control and cure. It some times involves the surrounding parts to such an extent as to occasion the most disastrous results; and especially is this the case when the disease assumes the form of tumor, and especially if it be malignant. But even the simple forms of disease of this cavity, such as inflammation and suppuration of, or prurient discharge from its lining membrane, will be found frequently to be of a most persistent character, not yielding easily to either local or systemic treatment. In this connection the occurrence of malignant growths is a condition most disastrous; this it is probable, however, only occurs when there is a systemic predisposition. The benign and malignant affections of the soft and hard parts are liable to occur either more or less remotely as a consequence of deeayed teeth.

